# Transformer Decoder-Based Enhanced Exploration Method to Alleviate Initial Exploration Problems in Reinforcement Learning

**DOI:** 10.3390/s23177411

**Published:** 2023-08-25

**Authors:** Dohyun Kyoung, Yunsick Sung

**Affiliations:** 1 Department of Autonomous Things Intelligence, Graduate School, Dongguk University–Seoul, Seoul 04620, Republic of Korea; dohyunkyoung@dgu.ac.kr; 2 Division of AI Software Convergence, Dongguk University–Seoul, Seoul 04620, Republic of Korea

**Keywords:** machine learning, reinforcement learning, pretraining, exploration, transformer-decoder

## Abstract

In reinforcement learning, the epsilon (ε)-greedy strategy is commonly employed as an exploration technique This method, however, leads to extensive initial exploration and prolonged learning periods. Existing approaches to mitigate this issue involve constraining the exploration range using expert data or utilizing pretrained models. Nevertheless, these methods do not effectively reduce the initial exploration range, as the exploration by the agent is limited to states adjacent to those included in the expert data. This paper proposes a method to reduce the initial exploration range in reinforcement learning through a pretrained transformer decoder on expert data. The proposed method involves pretraining a transformer decoder with massive expert data to guide the agent’s actions during the early learning stages. After achieving a certain learning threshold, the actions are determined using the epsilon-greedy strategy. An experiment was conducted in the basketball game FreeStyle1 to compare the proposed method with the traditional Deep Q-Network (DQN) using the epsilon-greedy strategy. The results indicated that the proposed method yielded approximately 2.5 times the average reward and a 26% higher win rate, proving its enhanced performance in reducing exploration range and optimizing learning times. This innovative method presents a significant improvement over traditional exploration techniques in reinforcement learning.

## 1. Introduction

Reinforcement learning, a prominent machine learning method, involves an agent receiving rewards for actions taken and selecting actions to maximize these rewards [1]. It has witnessed significant progress through the application of deep learning and finds extensive use in gaming [2,3,4,5], robot control [6,7,8,9], and autonomous driving systems [10,11,12,13]. Gaming, in particular, benefits from reinforcement learning as it allows visual recognition of game screens as states and a clear definition of rewards, making it more intuitively manageable. Consequently, the field of gaming has seen a wide range of reinforcement learning research employing deep learning techniques, achieving performance comparable to that of humans [14,15,16]. Subsequent studies have explored action execution methods to enhance the naturalness and evolution of AI characters in games, further increasing players’ interest [17,18,19].

Exploration is a crucial aspect of reinforcement learning [1]. Agents need to visit various states and identify the optimal actions to learn the optimal policy, which necessitates exploration. Traditional reinforcement learning achieves exploration by randomly selecting actions using the epsilon-greedy strategy. However, in scenarios with a high number of actions, the action space becomes large, leading to prolonged learning times. Thus, there is a need for a method that reduces the exploration range of the agent’s state space during the early stages of exploration.

Methods have been proposed to address the exploration range issue in reinforcement learning by leveraging expert data. One approach involves narrowing the exploration range through statistical measures based on expert data when a significant discrepancy exists between predicted and actual actions during the agent’s exploration and learning process [20]. However, this approach has limitations as it cannot learn about states or actions not included in the expert data. Another method initiates and learns episodes based on consecutive states included in the expert data [21], but it restricts exploration to states within or adjacent to the starting positions found in expert data. Combining expert data with replay memory is another approach [22], wherein expert data are stored in replay memory and used alongside the agent’s experience, prioritizing learning from the expert data. However, this method presents challenges such as the need for large replay memory capacity and the risk of overfitting to the expert data.

Methods utilizing expert data for pretraining [23,24,25] enhance the representation power of neural networks in reinforcement learning through the use of pretrained models. These models are then employed for reinforcement learning after pretraining on expert data. However, these methods primarily focus on improving sample efficiency and generalization capabilities by utilizing pretrained models from different domains, rather than directly addressing the initial exploration problem in reinforcement learning. In other words, they effectively learn image representations through pretraining and utilize them to select better actions during reinforcement learning. Furthermore, since these methods directly utilize pretrained models on expert data as deep learning models for reinforcement learning, they are susceptible to overfitting. Therefore, there is a need for a method that directly reduces the initial exploration range across the entire state space using expert data, while mitigating overfitting and improving action prediction accuracy through a pretrained model.

This paper proposes a method to address the issue of extended learning time during initial exploration in reinforcement learning by employing a transformer decoder pretrained through supervised learning with massive expert data. Initially, the agent’s actions are determined by the pretrained transformer decoder based on expert data. To prevent overfitting, once a certain level of learning is achieved, the epsilon-greedy strategy is employed to perform random actions and actions derived from the reinforcement learning model, facilitating policy learning. By learning the policy through expert data, the exploration range can be reduced while considering the entire state space. Additionally, using the transformer decoder as a pretrained model allows for the consideration of consecutive states while mitigating the long-range dependency problem encountered in models based on Recurrent Neural Networks (RNN). Therefore, in contrast to existing methods that primarily use pretraining to improve sample efficiency and generalization capabilities, the proposed method directly addresses the initial exploration problem in reinforcement learning. Furthermore, this method promotes learning and reduces the risk of overfitting by judiciously incorporating a pretrained model into the reinforcement learning process. The main contributions of the proposed method are as follows:

- Enhancing action prediction ability by leveraging the transformer decoder as a pretrained model.
- Reducing the exploration range while considering the entire state space, rather than limiting exploration to adjacent states included in the expert data.
- Mitigating overfitting, reducing the initial exploration range, and shortening the learning time by employing the pretrained model of the transformer decoder, which helps guide agent actions for a certain episode before the reinforcement learning model is employed.

The remainder of this paper is organized as follows. Section 2 discusses the related research pertaining to the proposed method. Section 3 provides a detailed explanation of the proposed method. Section 4 presents the learning environment and various experimental results. Finally, Section 5 summarizes the proposed method, highlights the experimental results, and discusses the anticipated benefits.

## 2. Related Works

This section presents various methods for addressing the exploration problem in reinforcement learning, focusing on those that incorporate probabilistic elements and utilize expert data.

### 2.1. Stochastic Exploration Methods in Reinforcement Learning

Traditional reinforcement learning often employs exploration methods that randomly select actions based on probabilities. The epsilon-greedy strategy [1,26] is one such approach that encourages exploration by choosing random actions with a fixed probability (epsilon, ε). In the initial learning stages, all options are explored through random action selection. As learning progresses, epsilon decreases, leading to the selection of the optimal action. However, when the number of actions is increased, the exploration range widens, which leads to extended training times.

The Boltzmann Exploration method [27,28] used in reinforcement learning involves probabilistically selecting actions. It generates a probability distribution for all possible actions an agent can perform and selects actions based on this distribution. Unlike the epsilon-greedy strategy, which selects actions randomly, the Boltzmann Exploration method assigns consistent probabilities to all actions and updates them as learning progresses, increasing the chances of selecting the optimal action. This method can enhance performance in the early learning stages. However, calculating the probability distribution for each action can be time-consuming when the number of actions is large.

The maximum entropy exploration method [29,30] incorporates an entropy bonus to balance exploration and exploitation in an agent’s decision-making process. The entropy bonus encourages the agent to experience a variety of actions, promoting exploration. This approach updates policies to maximize uncertainty and ensures equal selection of all actions. It maintains a balance between exploration and exploitation by considering the probability distribution of all possible actions and simultaneously maximizing rewards. However, calculating expected values for all possible actions increases computational complexity and may reduce learning efficiency if the agent continues to perform actions that yield small rewards.

The noise exploration method [31,32] introduces random noise into the network of the reinforcement learning model to facilitate exploration. By adding noise, the probability of performing exploratory behavior increases, allowing the agent to gain a variety of experiences. However, the consistency of the model may be compromised due to different action selections from the same state caused by noise, leading to generalization problems in the learning model. Additionally, the outcomes of actions and environmental rewards may become ambiguous, hindering reward prediction and the learning process.

The proposed method addresses the exploration range issue in the initial learning stages by using a pretrained model. Even with a large exploration range, the impact on the learning process can be effectively managed. Furthermore, the agent learns the behavior patterns of experts through expert data and the pretrained model, addressing the generalization problem in the reinforcement learning model and reducing learning time.

### 2.2. Exploration Methods Using Expert Data for Reinforcement Learning

Several methods utilize pretraining based on expert data to tackle the initial exploration problem in reinforcement learning. Silver et al. [33] achieved Grand Challenge-level performance in Go by combining the Monte Carlo Tree Search (MCTS) method with deep learning. However, this approach requires extensive computing resources and has long learning and inference times due to simulating all possible actions and making selections accordingly. While the Convolutional Neural Networks (CNN) used for pretraining are effective in learning spatial features, they struggle with understanding persistent situations or temporal changes.

Vecerik et al. [34] proposed a method to enhance performance in the robotics field by merging generative adversarial imitation learning (GAIL) with hindsight experience replay (HER). GAIL imitates expert behavior to learn the agent’s policy, while HER allows effective learning from failed attempts by reusing past experiences. However, as GAIL is based on the Generative Adversarial Networks (GAN), it may encounter mode collapse during the learning process.

Hester et al. [35] introduced a method for initializing and training the Deep Q-Networks (DQN) algorithm using expert data. This approach enables the agent to learn the optimal policy during the initial learning stages by incorporating expert data into the network’s updates. However, overfitting to expert data may occur if rewards are frequently provided for selecting expert data.

Nair et al. [36] proposed a solution to the initial exploration problem in reinforcement learning by combining Deep Deterministic Policy Gradient (DDPG) with expert data. This method applies DDPG updates to DQN based on expert data, leading to policy improvement through reinforcement learning. However, it requires two replay memories, which increases storage space requirements and limits the predictive ability of the pretrained model.

Aytar et al. [37] pretrained a model on video clips instead of previously played data to learn policies in complex environments. However, this approach increases the model’s complexity due to the use of video visual features and audio features for training.

The aforementioned methods directly incorporate expert data into the reinforcement learning model, which can lead to model dependency issues. In the proposed method, pretraining is conducted with expert data, and initial learning occurs through the pretrained model during reinforcement learning. After a certain amount of learning, the method transitions to traditional reinforcement learning exploration methods. This process helps prevent overfitting to the expert data. The initial training utilizes a transformer decoder, which considers consecutive states and mitigates long-term dependency issues, enabling accurate action prediction.

## 3. Advanced Exploration Method in Reinforcement Learning Using a Transformer Decoder

This section describes a method to address the issue of extended learning time resulting from the initial exploration issue in reinforcement learning. The approach involves training the agent’s policy using a pretrained transformer decoder during the initial exploration stages of reinforcement learning.

### 3.1. Overview

**Figure 1:**
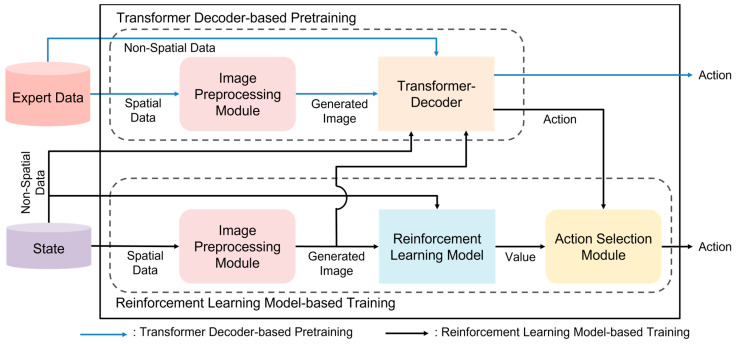
Transformer decoder-based enhanced exploration method.

The objective of the proposed method is to find the optimal actions for the agent through learning with a transformer decoder and reinforcement learning. The proposed method consists of two processes: transformer decoder-based pretraining and reinforcement learning-based Training. Figure 1 provides an overview of the proposed method.

During the transformer decoder-based pretraining phase, the image preprocessing module is employed to convert expert data into images. The pretrained transformer decoder, which is trained prior to reinforcement learning, then processes the generated images from spatial data and non-spatial data to derive actions.

The reinforcement learning model-based training process involves the image preprocessing module for generating images, a reinforcement learning model, and an action selection module for choosing actions. The state received from the environment contains both spatial and non-spatial data. Spatial data are transformed into images using the image preprocessing module. These images, along with the non-spatial data, serve as inputs to the reinforcement learning model. Actions are determined in the action selection module based on the output values from the reinforcement learning model, either through the pretrained transformer decoder or using the epsilon-greedy strategy.

### 3.2. Transformer Decoder-Based Pretraining

The transformer decoder-based pretraining process aims to provide actions for selection during the initial exploration of reinforcement learning by pretraining the transformer decoder using expert data. This section explains the processing of expert data and the learning structure in transformer decoder-based pretraining.

**Figure 2:**
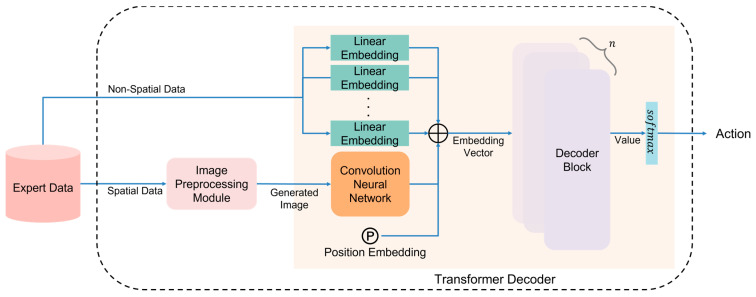
Transformer decoder-based pretraining process.

Figure 2 illustrates the transformer decoder-based pretraining process. The spatial data from the expert data are converted into images through the image preprocessing module. The specific method of generating images depends on the learning field or the information contained in the expert data, which will be elaborated in detail in the Experiment section. The transformer decoder comprises a CNN for deriving spatial features, several linear embedding layers, a positional embedding layer for representing location information, and *n* decoder blocks. The generated images and non-spatial data are directly input into the transformer decoder. The CNN within the transformer decoder transforms the images into spatial features, while the non-spatial data are divided and input into each linear embedding layer. The linear embedding layer converts the semantic information into fixed-sized vectors. The CNN feature and linear embedding vector are concatenated, and positional embedding is added. The resulting embedding vector is then fed into consecutive decoder blocks, and actions are derived through the Softmax applied to the final output. The decoder block is based on the model proposed by Radford et al. [38]. This model, known as GPT2, characteristically employs only the decoder structure of a transformer. It leverages the mechanism of self-attention and positional embeddings to capture the relationships between each element in the input, which aids in effectively learning contextual information. A decoder block of the proposed method is based on this architecture, tailored to reflect features derived from expert data.

The difference between the expert actions and the actions predicted by the transformer decoder is calculated using cross entropy. By minimizing this difference and deriving the probability distribution for each class, the model is trained to reduce the discrepancy.

### 3.3. Reinforcement Learning Model-Based Training

**Figure 3:**
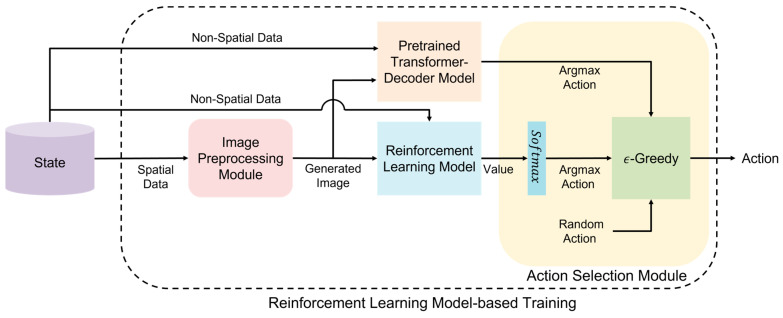
Reinforcement learning model-based training process.

The reinforcement learning model-based training process introduces the method of training the proposed approach through reinforcement learning. The policy update process is similar to traditional reinforcement learning. However, to address issues during the initial exploration phase, the pretrained transformer decoder model is utilized. The reinforcement learning model learns from the state received from the environment. Images generated using the spatial data of state and non-spatial data are input to train the reinforcement learning model, identical to the approach used in transformer decoder-based pretraining. Figure 3 provides a detailed depiction of the reinforcement learning model-based training process, focusing on the structure of the action selection module responsible for determining actions.

The values derived from the reinforcement learning model are used to select actions through the action selection module. This module chooses actions through one of three methods. First, if the hyperparameter δ(0 < δ < 1) is greater than $\frac{Episode}{N}$ ($\delta \geq \frac{Episode}{N}$), the action derived from the pretrained transformer decoder is chosen. Herein, Episode denotes the current episode, and N denotes the total episodes. The second and third methods involve either selecting an action randomly according to the epsilon-greedy strategy or selecting an action derived from the reinforcement learning model. As the episodes progress and $\frac{Episode}{N}$ exceed δ ($\delta <\frac{Episode}{N}$), actions are determined according to the epsilon-greedy strategy, a commonly used exploration method in traditional reinforcement learning. To mitigate the influence of outliers with large error values and stabilize learning, the Huber loss function [39] is used with the threshold λ set to 1.

Algorithm 1 presents pseudo code according to the training process of the reinforcement learning model in the proposed method, which is described mathematically as follows.

(1) $$s=\left(s^{+},s^{-}\right),\;\varphi =\xi \left(s^{+}\right),\;S=(\varphi ,\;s^{-})$$

State s consists of a pair of spatial data $s^{+}$ and non-spatial data $s^{-}$. At this point, the spatial data $s^{+}$ are transformed into an image φ through an image preprocessing module ξ.

The action a is selected differently based on the threshold δ representing the episode progression ratio and $\frac{Episode}{N}$. If $\delta \geq \frac{Episode}{N}$, a is derived through a pre-trained transformer decoder *P*. Conversely, if $\delta <\frac{Episode}{N}$, a is derived using an epsilon-greedy strategy. However, depending on the epsilon value ϵ(0<ϵ<1), a is chosen randomly, or a with the maximum Q-value is chosen. This can be expressed as follows:

(2) $$a=\left\{\begin{array}{c} \;\;\;P(S),\;\;if\;\;\delta \geq \frac{Episode}{N}\;\;\;\;\;\;\;\;\;\;\;\;\;\;\;\;\;\;\;\;\;\;\;\;\;\;\;\\ random\;action,\;\;if\;\;\delta <\frac{Episode}{N}\;and\;\epsilon >random\;value\\ \;\;\;\;\;\;argmax\;Q(S),\;\;if\;\;\delta <\frac{Episode}{N}\;and\;\epsilon <random\;value\;\;\;\; \end{array}\right.$$

The random value is compared to ϵ. If this value is lower than ϵ, a random action is taken. If not, the action with the maximum Q-value is executed.

When the agent performs the determined action, the reward $r_{t}(S_{t}$, $a_{t})$, for the t step, non-spatial data $s_{t+1\;}^{-}$, for the next state, and the next state image $\varphi_{t+1}$ are obtained and these values are stored in the replay memory M.

(3) $$M=\{\varphi_{t},s_{t}^{-},\;a_{t},r_{t},\;\varphi_{t+1},\;s_{t+1}^{-}\}$$

In this paper, we leveraged DQN as the reinforcement learning model. The replay memory M is initialized, and the Q-network is initialized with random weights θ. Moreover, the target network G is initialized using weights $\theta^{+}$ that are identical to random weights θ, and the pretrained transformer decoder model is initialized with pretrained weights $\theta^{L}$. The state s is separated into spatial data $s^{+}$ and non-spatial data $s^{-}$. During an episode, an image φ=ξ($s^{+}$) is generated based on spatial data. ξ is an image preprocessing module. If $\delta \geq \frac{Episode}{N}$, the action a is chosen using the pretrained transformer decoder P. As the episode progresses and $\delta <\frac{Episode}{N}$, an action a is selected based on the epsilon-greedy strategy, a common exploration method in traditional reinforcement learning. By performing the determined action $a_{t}$, reward $r_{t}$, next state non-spatial data $s_{t+1}^{-}$, and the next state image $\varphi_{t+1}$ are obtained. The values of $\varphi_{t},\;s_{t}^{-},\;a_{t},r_{t}$,$\varphi_{t+1},\;s_{t+1}^{-}$ are stored in the replay memory M. Mini-batch samples are randomly selected from the replay memory. Depending on whether the state is terminal or non-terminal, the reward that the agent receives, and the Q-value are added to calculate $y_{j}$. By using the Huber loss, the weights θ of the Q-Network are updated to reduce the loss between $y_{j}$ and the Q-value predicted by the DQN model. Finally, the update of the target network is performed every Cth episode, and this process is repeated to the Nth episode.
**Algorithm 1** Pretrained Transformer Decoder-based exploration method in DQN   **Initialize**  Replay memory M
  Q-Network Q ← random weights θ  Target Network G ← weights $\theta^{-}=\theta$
  Pretrained Transformer Decoder *P* ← pretrained weights $\theta^{L}$  **For** episode = 1, *N*
**do**  Get state s and separate s into spatial data $s^{+}$ and non-spatial data $s^{-}$   Generated image φ=ξ($s^{+}$)   **for** step *t* = 1, *T*
**do**  if $\delta \geq \frac{Episode}{N}$, select action $a_{t}=P\left(\varphi_{t},\;s_{t}^{-}\right)$  else,      with probability ϵ, select a random action $a_{t}$      otherwise, select action $a_{t}$ = ${max}_{a}Q^{*}$($\varphi_{t},s_{t}^{-},a$; θ)    Execute action $a_{t}$ and get reward $r_{t}$, next state image $\varphi_{t+1},$ and next state non-spatial data $s_{t+1}^{-}$    Store transition ($\varphi_{t}$, $s_{t}^{-}$, $a_{t}$, $r_{t}$, $\varphi_{t+1}$, $s_{t+1}^{-}$) in M    Sample random minibatch of transitions ($\varphi_{j},s_{j}^{-},a_{j},\;r_{j},\;\varphi_{j+1},\;s_{j+1}^{-}$) from M    Set $y_{j}$ $=\left\{\begin{array}{c} r_{j}\;\;\;\;\;\;\;\;\;\;\;\;\;\;\;\;\;\;\;\;\;\;\;\;\;\;\;\;\;\;\;\;\;\;\;\;\;\;\;\;\;\;\;\;\;\;\;\;\;\;\;\;\;\;\;\;\;\;if\;terminal\;at\;step\;j+1\;\;\;\;\;\;\;\;\;\;\;\;\;\;\;\\ r_{j}+\gamma {max}_{a^{\prime }}G\left(\varphi_{j+1},s_{j+1}^{-},a^{\prime };\theta^{-}\right)\;\;\;\;\;\;\;\;\;\;\;\;\;otherwise\;\;\;\;\;\;\;\;\;\;\;\;\;\;\;\;\;\;\;\;\;\;\;\;\;\;\;\;\;\; \end{array}\right.$    Perform a gradient descent step on    $\left\{\begin{array}{c} \frac{1}{2}{\left(y_{j}-\left(\varphi_{j},s_{j}^{-},a_{j};\theta \right)\right)}^{2}\;\;\;\;\;\;\;\;\;\;\;\;\;\;\;\;\;\;\;if\;\;\left|y_{j}-Q\left(\varphi_{j},\;s_{j}^{-},\;a_{j};\theta \right)\right|\leq \lambda \\ \lambda \left(\left|y_{j}-Q\left(\varphi_{j},s_{j}^{-},a_{j};\theta \right)\right|-\frac{1}{2}\lambda \right)\;\;\;\;\;\;\;\;\;\;\;\;\;\;\;\;\;\;\;\;otherwise\;\;\;\;\;\;\;\;\;\;\;\;\;\;\;\;\; \end{array}\right.$
    with respect to the Q-Network parameters θ   **end for**  Every *C* episodes reset G=Q  **Until** episodes <N


## 4. Experiment

This section introduces the experimental environment, data processing for the experiment, and experimental results. The Experimental Environment section reports definitions and values of various hyperparameters and settings for the experiment. The Experimental data section explains the preprocessing of the data for pretraining the transformer decoder. In the experimental results section, we present the experimental process of the pretrained transformer decoder and compare the performance of the traditional reinforcement learning model, DQN, with the proposed method.

### 4.1. Experimental Environment

In this paper, the experiments are conducted in a game environment. The process of pretraining the transformer decoder uses expert data for training and testing, and we validated the transformer decoder’s accuracy of action prediction for pretraining. The process of conducting reinforcement learning involves comparing the performance of the traditional DQN method and the proposed method. Table 1 presents the learning parameters used in the transformer decoder for pretraining as explained in the proposed method, the traditional DQN, and the DQN using the pretrained transformer decoder, which is our proposed method.

**Figure 4:**
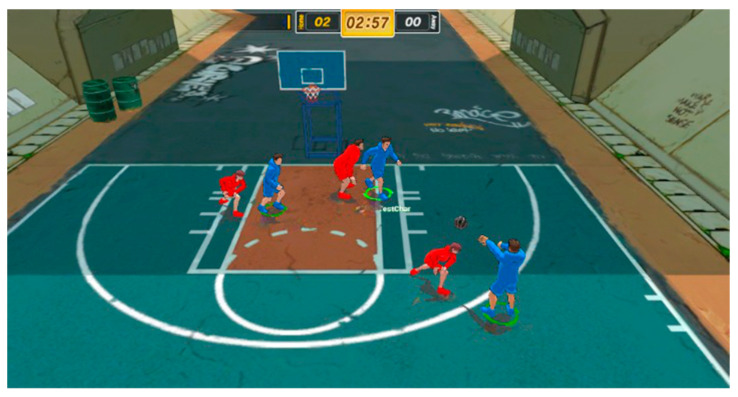
Screenshot of FreeStyle1 simulator.

This paper used the simulator of FreeStyle1, an online game from Korean Joycity Corp., as the experimental environment. FreeStyle1 is a 3-versus-3 basketball game that with a total runtime of 4 min. Unlike regular basketball games, it uses a half-court measuring 15 (m) × 11 (m). If the basketball does not go out of court and the offensive team does not shoot within 20 s, it is treated as a violation and the teams switch positions. The team with the higher score at the end of the 4-min game wins. The blue agents comprise the Home Team, whereas the red agents comprise the Away Team. Figure 4 presents a screenshot of the FreeStyle1 simulator.

Training and testing were performed on a Windows 10 system with a 12-core Intel i9-12900KF and an NVIDIA GeForce RTX 3080 TI (12 GB). Python 3.7 was used as programming platform, and we used the deep learning library TensorFlow 2.10 to enhance the computational efficiency.

### 4.2. Experimental Data

The proposed method requires expert data to train the transformer decoder. In this paper, the expert data are collected by the actual play of FreeStyle1 considering the quality of plays and allowing it to be considered as ground truth data. Approximately 15,000 expert data points were collected per game, amounting to a total of about 60,000 data points from four games. Among these 60,000 data points, 50,000 data points were used as training data, and the remaining 10,000 data points were used as validation data. The data derived from a single frame are shown in Table 2.

To train using expert data, each data point was labeled according to the corresponding action. There were 14 actions in total, and these were matched according to the MainState of each agent. The labeling of actions is presented in Table 3; “Attack” denotes the state of possessing the ball, “Offense” signifies the situation wherein the team is on the offense but does not possess the ball, and “Defense” signifies the defensive state.

**Figure 5:**
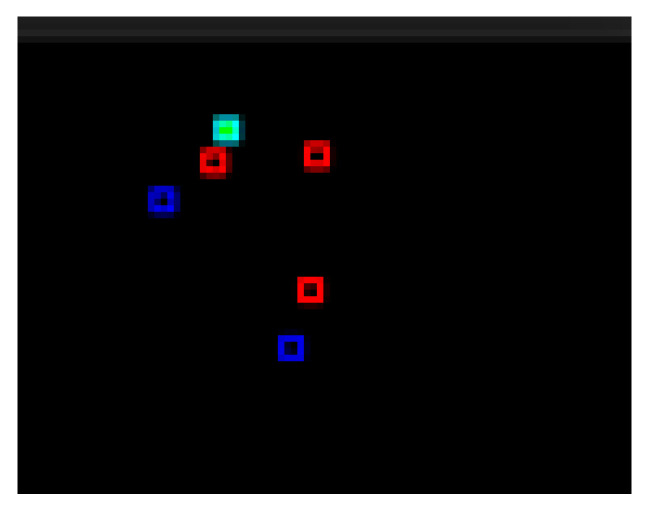
Generated image through the Image preprocessing module.

As shown in Figure 4, due to the complex environment and the issue of partial observability wherein the entire image cannot be observed in the FreeStyle1 Simulator images, a process of generating images using expert game state data is needed. In this paper, images were generated using only spatial data from the expert data and state. Spatial data involved the WhoAttack, Ball, HomeTeam, and AwayTeam data from Table 2. For generating images, we utilized the absolute coordinates (x, y) of each agent per team and the ball. The entire image was created with a size of 39 × 51 × 3. Each agent was drawn as a rectangle with only a border. However, the learning agent and ball were distinguished using a full rectangle. The attacking team was represented by a blue rectangle, the defensive team was represented by a red rectangle, and the ball was denoted by a green rectangle. The rectangle sizes for the current attacking team, defensive team, and ball representation were identical at 3 × 3. Although image information can be lost during training if the image is small, the images are simple and do not contain considerable information as they only represent the agents’ locations. Thus, the images were created at a small size for optimization. Figure 5 displays the image generated via the game image preprocessing module.

In the proposed method, BallClear, HomeTeam Position, HomeTeam MainState, AwayTeam Position, and AwayTeam MainState data are used as non-spatial data and are inputted into each linear embedding layer. Data other than spatial and non-spatial data do not affect learning and hence are not used.

### 4.3. Experimental Results

**Figure 6:**
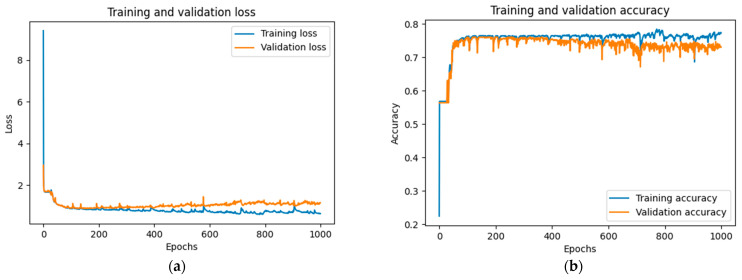
The training and validation result graphs of the Transformer Decoder in the Transformer Decoder-based pretraining process is as follows: (**a**) the training and validation loss graph of the Transformer Decoder; (**b**) the training and validation accuracy graph of the Transformer Decoder model.

To measure the performance of our proposed method, a pretrained transformer decoder is needed. The training and validation results of this pretrained transformer decoder are presented in Figure 6. The entire training process required 1000 epochs. Figure 6a displays the loss graph for both training and validation data, and Figure 6b shows their corresponding accuracy graphs. As shown in Figure 6a, the transformer decoder experienced an initial training loss of 9.54 and validation loss of 2.9. After approximately 200 epochs, these values dropped to around 1.24 and 1.4, respectively. However, after about 300 epochs, the losses began to increase again. Therefore, we chose the results obtained from training up to 300 epochs.

**Figure 7:**
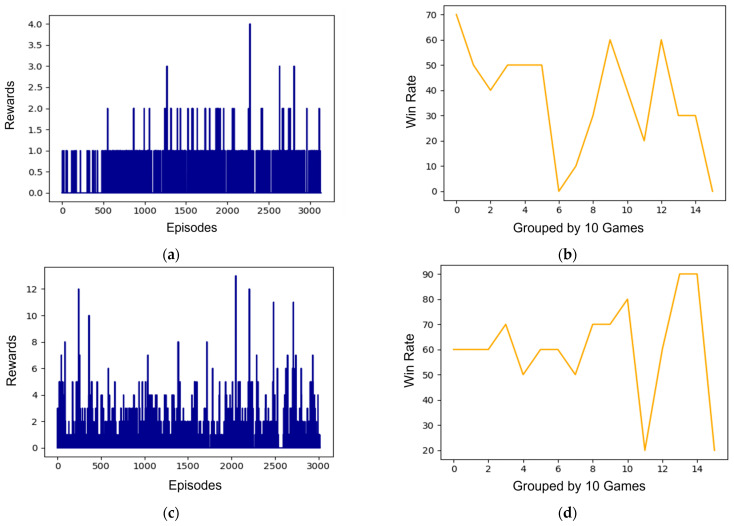
The result graphs of the traditional DQN and the proposed method are as follows: (**a**) the reward graph of the traditional DQN; (**b**) the win rate graph of the traditional DQN; (**c**) the reward graph of DQN applying the proposed method; (**d**) the win rate graph of DQN applying the proposed method.

Figure 7 presents the results of an experiment comparing the learning performances of a DQN with the proposed pretrained transformer decoder and a traditional DQN. Figure 7a,c shows reward graphs per episode of the traditional DQN and the proposed method, respectively. Additionally, Figure 7b,d graphs the win rate per ten games of the traditional DQN and the proposed method. As per Figure 7a, the initial rewards are approximately one due to random actions, and although they increased over time, the difference was minor. This indicates that increasing rewards significantly will take considerable time, as finding optimal actions requires extensive exploration. Figure 7b represents the win rate for every ten games, with draws being counted as wins. Owing to the coincidence of random actions, the win rate was high in the initial training phase and then gradually dropped. It remained at approximately 30–40% on average, suggesting that learning was not effectively accomplished, thereby failing to result in winning actions. In Figure 7c, it can be observed that the proposed method, which employs actions trained by the Transformer decoder in the win rate graph, receives high rewards from the early stages of training. Although the rewards are received irregularly, this is not problematic as it can vary depending on the state. It is evident that the proposed method finds optimal actions while receiving rewards, in contrast to the traditional DQN. The DQN using the proposed method in Figure 7d achieved an average win rate of 60–70%, showing higher peak and average win rates compared to the conventional DQN. With the win rate reaching up to 90%, this proves the efficacy of using a DQN with pre-training via a Transformer decoder over the traditional DQN.

The experimental results could be considered to be positive if the graph had a consistent upward trajectory in the Figure 7. However, given the probabilistic nature of scoring in basketball, maintaining this consistency proves challenging. Nonetheless, when comparing the graphs, it can be stated that the proposed method has learned effective strategies such as timing the shot for a successful score. The qualitative analysis of the experimental results can be summarized as follows: first, in the results of the traditional DQN, the initial rewards being low is due to the randomness of the actions, and the almost no change in rewards indicates that a broad range of exploration is needed to find the optimal actions. Second, the fact that the win rate of the traditional DQN remains at around 30–40% on average of the traditional DQN suggests that the learning is not effective. Third, the fact that the proposed method achieves high rewards in the early stages of training demonstrates its ability to find optimal actions through rewards. Finally, the achievement of a higher average win rate by the proposed method validates the effectiveness of pretraining through the transformer decoder.

**Figure 8:**
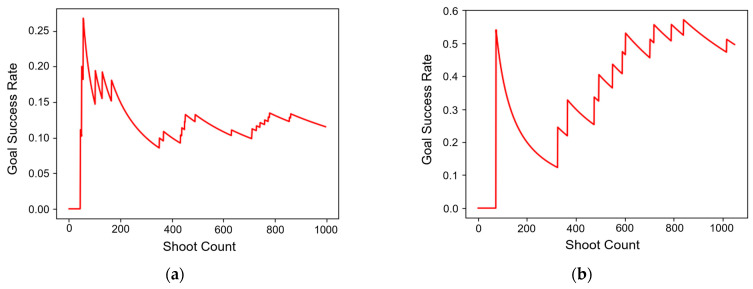
The additional result graphs of the traditional DQN and the proposed method are as follows: (**a**) the goal success rate graph of the traditional DQN; (**b**) the goal success rate graph of the proposed method.

For further comparison of the proposed method and traditional DQN, Figure 8 shows the number of goals scored per shot. An important point to note here is that indiscriminate shooting does not necessarily yield positive results; it is more advantageous to shoot in situations or positions that have a high probability of scoring. On the graph, a value closer to 1 signifies a higher likelihood of scoring per shot. Figure 8a shows the results of the traditional DQN. Initially, the scoring probability was around 0.25, indicating that out of ten attempted shots, about two to three would successfully score. However, as the training progressed, this rate fell to around 0.1 and thereafter remained between 0.1 and 0.15. This suggests that the model either failed to attempt shots in optimal scoring situations or shot in circumstances where it would have been better not to. Figure 8b shows the results of our proposed method. While the initial scoring ratio was over 0.5, it similarly plummeted to 0.1. However, unlike the traditional DQN, the scoring ratio of our method subsequently rose back up to approximately 0.5 as training continued. In conclusion, the proposed method, in contrast to the traditional DQN approach, attempts shots not indiscriminately, but in situations that are more likely to result in successful goals.

**Figure 9:**
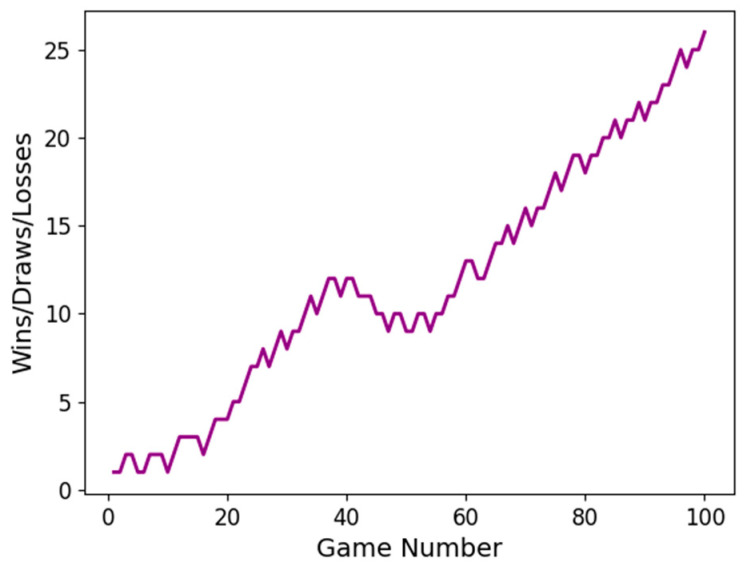
The wins/draws/losses accumulated graph of the DQN applying the proposed method.

Additionally, Figure 9 represents the cumulative game results of the DQN with the proposed method applied against the traditional DQN. A total of 100 games were played, and the proposed method resulted in 46 wins, 34 draws, and 20 losses, which is 26% better in terms of win rate.

## 5. Discussion

The proposed method does not modify the reward and penalty structure used in the underlying DQN model. Instead, it alters the action selection mechanism during the initial exploration phase. Regardless of whether an action is chosen based on the pretrained transformer decoder or according to the epsilon-greedy strategy, the received reward or penalty remains unaffected. In this way, our method maintains the inherent structure of the reinforcement learning process while striving to improve initial exploration problem and model performance.

Our method relies on the availability of reliable expert data. Therefore, in cases where such data are not available or is unreliable, the application of our method could be limited. Additionally, our method assumes that the overall situation can be encapsulated in data, some of which can be expressed as images. For example, the coordinates of the agents may be requested. Thus, in scenarios where such data representation is not feasible, the implementation of our method might not be suitable.

In this paper, we use expert data to guide the initial exploration, which allows us to reduce the exploration in reinforcement learning and potentially shorten the learning time. However, a potential downside of our method is that if the quality of the expert data, which we assume as ground truth, is not good, then a lot of time may be spent in exploration. This may require more exploration to find the optimal action compared to traditional reinforcement learning methods.

The proposed method performs well with structured data like images. However, to apply this method to more complex data like natural language, additional considerations would be necessary. For instance, we would have to use specific techniques for processing language (like word embeddings or deformed model) to transform the data into a form that our model can handle. We plan to explore these ideas further in our future work.

Future work could extend upon the proposed method by applying it to various other reinforcement learning algorithms like Proximal Policy Optimization (PPO) or Actor-Critic methods. These algorithms, which involve policy gradient methods, could potentially benefit from an initial policy derived from our method. The proposed method in this paper can be applied across various fields. In healthcare, it could potentially be applied to predictive healthcare models. Using historical patient data or sensor data as expert data, it could learn to predict disease progression or patient outcomes, informing treatment strategies. Similarly, in finance, the proposed technique could be employed in optimizing trading strategies, where historical trades could be treated as expert data. It could potentially learn from the most successful past actions to suggest profitable future trades. We hope that our method will be useful in a variety of scenarios represented by these examples.

## 6. Conclusions

This paper presents a method to address the challenge of initial exploration in reinforcement learning by utilizing a transformer decoder. The proposed approach involves two stages: pretraining the transformer decoder using expert data and subsequent training using reinforcement learning. Specifically, actions are selected using the pretrained Transformer decoder, and the action policy is updated during the initial exploration phase of reinforcement learning based on the threshold δ. Subsequently, the traditional epsilon-greedy strategy is employed for learning. This method effectively mitigates the prolonged learning times associated with reinforcement learning, which result from extensive exploration during the initial phase, by leveraging a transformer decoder pretrained with expert data. The experiment was conducted using the basketball game FreeStyle1, where a comparison was made between the traditional DQN model and a DQN model augmented with the pretrained transformer decoder. When trained for the same number of episodes, the DQN with the transformer decoder achieved a reward per episode that was approximately 150% higher. Moreover, in games where three agents utilized the traditional DQN and the remaining three employed the DQN with the transformer decoder, the latter exhibited an approximately 26% higher win rate.

Through this paper, we expect to arouse people’s interest by creating an AI that moves according to the player’s actions rather than mechanically. Additionally, it can be applied not only to the game field but also to various fields such as sensor data in health care, finance, and education.

## Figures and Tables

**Table 1:** Parameters for training the proposed method and baseline DQN model.

Hyperparameter	Pretrained Transformer Decoder	DQN	The Proposed Method
Image Size	(39, 51, 3)	(39, 51, 3)	(39, 51, 3)
Sequence Length	64	-	-
Attention Heads	16	-	-
Decoder Blocks	16	-	-
Hidden Dimension	768	-	-
Delta (δ)	-	-	0.3
Batch Size	64	64	64
Learning Rate	$1\times 10^{-5}$	$1\times 10^{-4}$	$1\times 10^{-4}$
Training Epochs	1000	-	-
Optimizer	Adam	Adam	Adam
Loss Function	Cross Entropy	Huber	Huber
Total Episode	-	3000	3000

**Table 2:** Data Contained in One Frame Information.

Data	Data Type	Description
GameState	Int	State of current game
BallClear	Int	Ball clear status for each team
HomeScore	Int	Home team’s current score
AwayScore	Int	Away team’s current score
GameTime	Int	Remaining time in the game (s)
WhoAttack	Int	Indication of the attacking and defending team
Ball	Float	Coordinates of the ball (x, y, z)
Lim	Float	Coordinates of the Lim (x, y, z)
HomeTeam Position	Int	Position for each home team character
HomeTeam GetBall	Int	Possession of the ball for each home team character
HomeTeam MainState	Int	Main state for each home team character
HomeTeam Coordinates	Float	Coordinates for each home team character (x, y, z)
AwayTeam Position	Int	Position for each away team character
AwayTeam GetBall	Int	Possession of the ball for each away team character
AwayTeam MainState	Int	Main state for each away team character
AwayTeam Coordinates	Float	Coordinates for each away team character (x, y, z)

**Table 3:** Labeling of Actions.

Action	Index
Up	0
UpLeft	1
Left	2
LeftDown	3
Down	4
DownRight	5
Right	6
RightUp	7
Shoot Fake	8
Shoot (Attack)/Rebound (Offense)/Block (Defense)	9
Pass (Attack)/Pass Call (Offense)/Steal (Defense)	10
Breakthrough (Attack, Offense)/Shootmark (Defense)	11
Backdown (Attack)/Screen (Offense)/Faceup (Defense)	12
Stand	13

## Data Availability

Not applicable.
